# How Is the Spectrum of Sarcoma Surgery Assessed?

**DOI:** 10.3390/cancers15041305

**Published:** 2023-02-18

**Authors:** Carlo Theus-Steinmann, Georg Schelling, Philip Heesen, Stefan Breitenstein, Mario F. Scaglioni, Bruno Fuchs

**Affiliations:** 1 Swiss Sarcoma Network (SSN), Luzerner Kantonsspital (LUKS), Kantonsspital Winterthur (KSW), 6000 Luzern, Switzerland; 2 Faculty of Health Sciences and Medicine, University of Luzern, 6002 Luzern, Switzerland

**Keywords:** sarcoma, multidisciplinary team/MDT, sarcoma surgery, orthopedic oncology, real-world data, interoperable digital platform, exposure, experience

## Abstract

**Simple Summary:**

Sarcoma surgery is the cornerstone of sarcoma therapy, which is organized highly multidisciplinarily. The critical determinant of tumor control depends on the experience of the multidisciplinary team (MDT), in which sarcoma surgery plays a pivotal part. In this study, an interoperable digital platform on sarcoma surgery was established to assess its spectrum based on a single sarcoma surgeon over one decade as a pilot. Being used at large scale, this platform may become an indispensable instrument to assess the contributions of sarcoma surgery within an MDT to tailor personalized patient quality care in the future.

**Abstract:**

Purpose: To meet the challenges of the precision medicine era, quality assessment of shared sarcoma care becomes pivotal. The MDT approach is the most important parameter for a successful outcome. Of all MDT disciplines, surgery is the key step to rendering sarcoma patients disease free; therefore, defining its spectrum is critical. To the best of the authors’ knowledge, a comprehensive interoperable digital platform to assess the scope of sarcoma surgery in its full complexity is lacking. Methods: An interoperable digital platform on sarcoma surgery has been created to assess the clinical exposure, tumor characteristics, and surgical settings and techniques applied for both resections and reconstructions of sarcomas. Results: The surgical exposure of an individual surgeon over time served as a pilot. Over the study period of 10 years, there were 723 sarcoma board/MDT meetings discussing 3130 patients. A total of 1094 patients underwent 1250 surgical interventions on mesenchymal tumors by one single sarcoma surgeon. These included 615 deep soft tissue tumors (197 benign, 102 intermediate, 281 malignant, 27 simulator, 7 metastasis, 1 blood); 116 superficial soft tissue tumors (45 benign, 12 intermediate, 40 malignant, 18 simulator, 1 blood); and 519 bone tumors (129 benign, 112 intermediate, 182 malignant, 18 simulator, 46 metastasis, 14 blood, and 18 sequelae of first treatment). Detailed types of resections and reconstructions were analyzed. Conclusions: An interoperable digital data platform on sarcoma surgery with transparent real-time descriptive analytics is feasible and enables large-scale definition of the spectrum of sarcoma surgery to meet the challenges of sarcoma precision care in the future.

## 1. Introduction

Sarcoma treatment includes various disciplines and is carried out by so-called multidisciplinary teams (MDTs). MDTs represent the cornerstone for the quality of sarcoma care [1,2,3,4,5]. Recently, quality indicators of global sarcoma care were reported [6]. Quality of sarcoma care is greatly dependent on various disciplines collaborating under one roof and its associated infrastructure and processes, as well as an adequate surgery and the surgical margins achieved thereby [7]. The latter, in turn, depends on the experience of the surgeon and his team and the complexity of the procedure. Of all the involved disciplines, surgery is the most important pillar to render a patient disease free and, hence, a surgeon’s experience plays a pivotal role; for this reason, the quality of surgery deserves particular attention [8]. Counting the number of surgeries alone serves at best as a surrogate but does not reflect per se the quality of surgery or the surgeon’s experience. For example, the surgical procedure of an Ewing sarcoma of the great toe differs greatly from that on the pelvis, as does the biology of the wide array of sarcoma entities representing different diseases. A sarcoma surgeon, therefore, is not only technically skilled but also understands the biology and various treatment aspects of the disease, including the process of performing longitudinal follow-up of the patients over time [9,10]. Most importantly, the sarcoma surgeon is capable of assembling a multidisciplinary team for sarcoma care, specifically for the wide and complex spectrum of surgical resections and reconstructions [9]. However, before the complexity or indicators of quality for sarcoma surgery are defined specifically, the surgical spectrum needs to be described by outlining the role of a sarcoma surgeon. Sarcomas may arise in any part of the entire body, thereby requiring an entire spectrum of surgical techniques, which one single surgeon in present times is unable to cover. Sarcoma surgery may include not only the resection of the tumor alone, but also subsequent reconstructions, adding another level of surgical complexity. Although sarcoma resection is driven by the biology of the lesion, which is most often independent of the anatomic location, reconstruction is highly site dependent because surgical techniques vary greatly depending on the anatomical locations. For these reasons, sarcoma surgery needs to be organized in a highly transdisciplinary fashion by personalizing each sarcoma surgery specifically to each patient’s situation, which does need to be taken into account when defining the complexity or also the quality of sarcoma surgery.

Health care cost explosion and the emerging skills shortage require the development of a novel ecosystem, moving away from a legacy system to a value-based system, in which the patient’s value is defined by the quality and outcome divided by the total costs over the full care cycle [4,11,12,13,14]. Moreover, from this economic perspective, the definition of quality of sarcoma care is indispensable. Sarcoma surgery shows a great level of complexity, which, in turn, is intimately related to the experience of the respective surgeon [15,16]. Defining the spectrum of sarcoma surgery is paramount to then defining the complexity of a procedure, but also for personalized teaching of the next generation of sarcoma surgeons and for continuous education purposes, as well as ultimately ascertaining the quality in every day practice and patients’ safety within an MDT. Defining the spectrum of sarcoma surgery may also assist in addressing the geography model of care by the regionalization of our patients, depending on patient- and disease-based parameters of sarcoma and the establishment of integrated practice units. Above all, it may make it possible to revisit the current reimbursement system in many countries without the capacity to mirror the specific scope of sarcoma surgery adequately using commonly available clinical information systems [4,11,12,13,14]. Therefore, challenges include the assessment of the various types and the technical aspects of surgical procedures using structured data on a respective interoperable digital platform [17].

To the best of the authors’ knowledge, there are no reports on how to assess and report on the spectrum of sarcoma surgery within an MDT. Because most of the clinical information systems in hospitals are not designed for the detailed search of sarcoma-surgery-specific aspects, we designed a novel web-based interactive real-world-time (RWDT) interoperable digital platform on sarcoma surgery to assess, identify, and analyze the spectrum of sarcoma surgery to meet the challenges of the precision medicine era.

## 2. Materials and Methods

A set of parameters including all single steps of all types of sarcoma surgeries was assembled [18]. As a prototype, this list was then applied on all surgeries of mesenchymal tumors performed by one single surgeon over a 10-year period. Registration was performed using the AdjumedCollect “Interoperable digital platform on Sarcoma Surgery” (Adjumed Services, AG, Zurich, Switzerland, http://www.adjumed.com/ (accessed on 30 November 2022)). The AdjumedAnalyze tool (Adjumed Services AG, Zurich, Switzerland) can be used for basic statistics, such as combinations of parameters, and allows for the extraction of data. The individual scores were calculated later in Microsoft Excel (Microsoft Corporation, Redmond, WA, USA).

**Figure 1:**
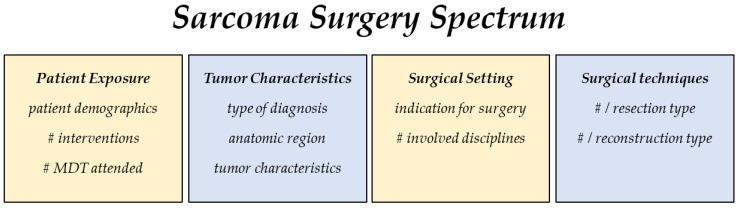
The exposure to sarcoma surgery is assessed in the following 4 categories: patient exposure, tumor characteristics, surgical setting, and surgical techniques applied. # number of.

The parameters to describe the sarcoma surgery spectrum include four main categories: clinical patient exposure, tumor characteristics, surgical settings, and techniques (Figure 1).

## 3. Results

### 3.1. Patient Exposure

**Figure 2:**
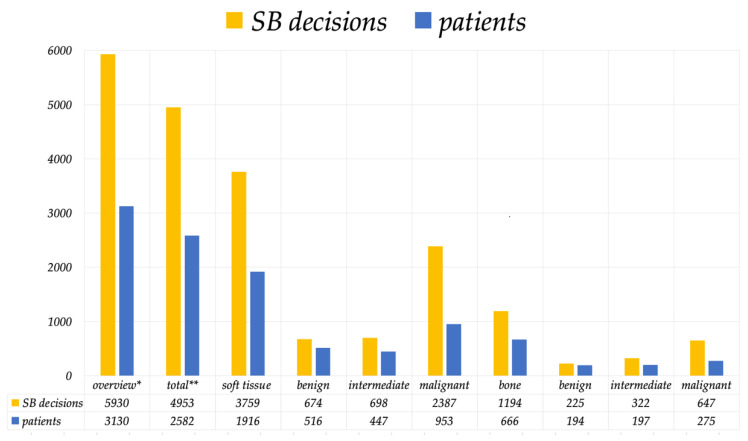
This figure summarizes the number of sarcoma board decisions and patients over a 10-year period. * All evaluations of mesenchymal tumors ** Exclusive metastasis, carcinoma, lymphoma, leukemia, myeloma, and tumor simulator.

Over a 10-year period of time, there were 723 MDT or sarcoma board meetings, in which 3130 patients were discussed, and 5930 sarcoma board decisions were made (Figure 2). This averages a total of 313 patients and 593 sarcoma board decisions per year.

**Figure 3:**
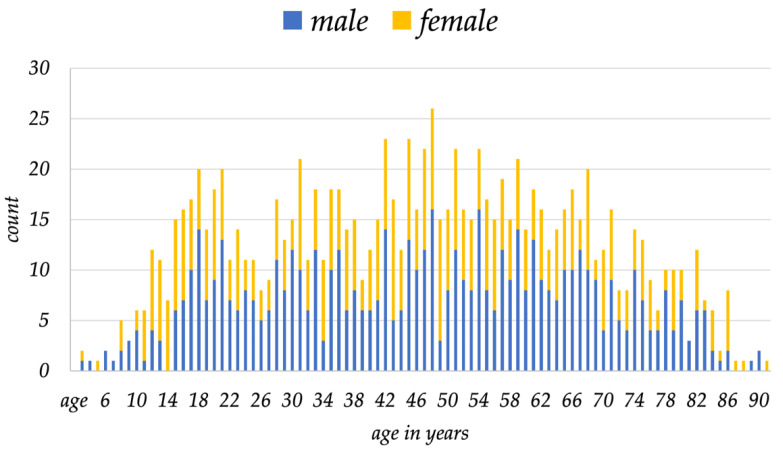
Distribution of gender and age over time of all patients included in this study is shown.

During the same 10-year period, one single surgeon performed a total of 1250 surgical interventions on mesenchymal tumors in a total of 1094 patients, who are the subjects of this analysis. There were 484 females and 610 males, with a mean age at surgery of 46.1 years (range: 1 to 91 years) (Figure 3).

### 3.2. Tumor Characteristics

In all 1094 patients, there were 628 soft tissue tumors, 339 bone tumors, and 44 metastases treated by surgery. The exact diagnoses are summarized in Table 1.

**Figure 4:**
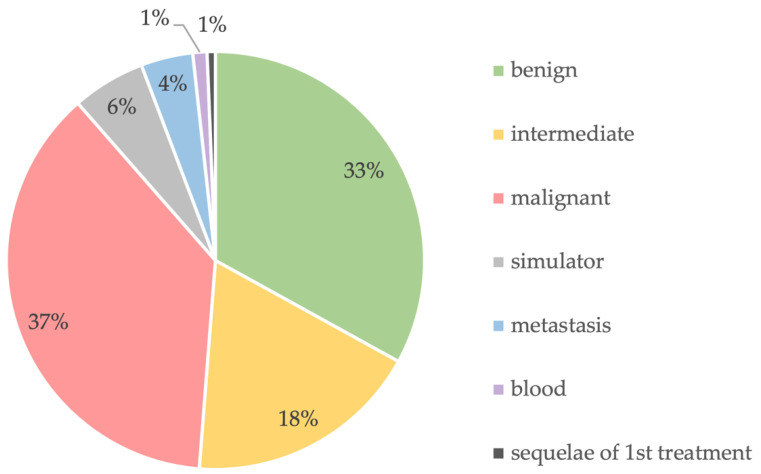
Biological diagnosis of the lesions.

**Figure 5:**
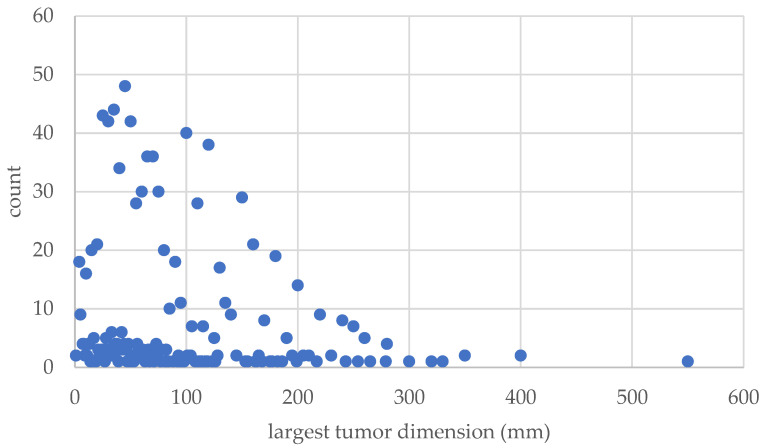
This diagram shows the number and size of the tumors.

Of these tumors, there were 361 benign, 199 intermediate, 409 malignant (34 G1, 85 G2, and 289 G3, respectively), 62 sarcoma simulators, 44 metastases, 12 blood, and 8 sequelae of prior therapy (Figure 4). In total, 266 underwent preoperative radiation therapy, 63 underwent postoperative radiation therapy, and 126 underwent neoadjuvant chemotherapy. The mean size of the tumors averaged 80.3 mm (range: 1 to 550 mm) (Figure 5).

Of these interventions, 615 concerned the deep soft tissue (197 benign, 102 intermediate, 281 malignant, 27 simulator, 7 metastasis, 1 blood); 116 cases concerned the superficial soft tissue (45 benign, 12 intermediate, 40 malignant, 18 simulator, 1 blood); and 519 concerned the bone (129 benign, 112 intermediate, 182 malignant, 18 simulator, 46 metastasis, 14 blood, and 18 sequelae of first treatment). From head to toe, 13 of all interventions were located in the head/neck/face region, 301 in the upper extremity, 87 in the torso/chest/abdomen, 159 in the pelvis, and 690 in the lower extremity.

### 3.3. Surgical Settings

The indication for surgery is an important parameter to describe the complexity of the patient cohort. Of all 1250 surgical interventions, in 996 cases (79.7%), surgery was indicated for the first time. In total, 56 cases (4.5%) had prior whoops surgery, and 17 cases (1.4%) presented with a pathological fracture. There were 52 first revision surgeries (4.2%) for any cause, and 41 second or more revision surgeries (3.3%). In total, 45 cases (3.6%) underwent surgery for a local recurrence (independent of whether the cases were of primary or referred patients), and 35 surgeries (2.7%) were indicated for more than 2 local recurrences. In total, eight surgeries (0.6%) were performed for other reasons, such as three for regional metastasis, two for systemic recurrence (one intraabdominal and one spine), two for removal of osteosynthesis material after fracture care, and one for a local progression of a multiple myeloma.

The definition of the surgical margin is not uniformly accepted [15], and the surgeon’s judgement on the resected margin does not necessarily reflect the pathologist’s opinion, nor the shared decision process of the MDT/sarcoma board. In the presented series, the surgeon defined wide/adequate margins in 933 surgeries (95.9%), in 18 marginal (1.8%) surgeries, and 23 intralesional (2.4%) surgeries, and margin status was not applicable in 276 surgeries because there was no sarcoma.

Of all surgeries, 875 were carried out by the sarcoma surgeon alone (70%), whereas 309 surgeries were performed with an expert from another discipline (24.7%), 53 surgeries with 2 additional disciplines (4.2%), and 4 surgeries each with 4 and 5 additional disciplines (0.3% each). In one surgery, namely a forequarter amputation with chest wall resection due to a post-irradiation UPS (undifferentiated pleomorphic sarcoma) infiltrating the brachial plexus, a total of 7 different disciplines were involved (sarcoma, orthopedics, chest, vascular, neuro, plexus, and reconstructive surgery).

### 3.4. Surgical Techniques

Surgical techniques focus on both resection and reconstruction. Resection techniques depend on the anatomic location and the specific structures that need to be removed. In this series, besides tumor resection itself, additional resection included 1800 surrounding and different types of soft tissues and 489 bone resections, 11 chest/thorax resections, 19 abdominal structures, and 106 sequelae of first treatment (e.g., débridement or prosthesis related resections).

Reconstructions after tumor resection were necessary in a total of 640 cases. They consisted of 319 bony reconstructions, including 94 prostheses, 84 allografts, 79 ORIF (incl. 18 pedicle screws/rods/cages), 24 autografts, 20 cementations (incl. 2 cement spacers), 4 arthrodeses, 2 gore-tex mesh, 1 distraction osteogenesis, and 11 other bone reconstructions (e.g., external fixator or Tikhoff–Linberg hanging bridge reconstruction).

Soft tissue reconstruction consisted of 38 tendon/ligaments, 70 neurovascular structures (56 vessels and 14 nerves), 16 abdominal, and 11 chest wall reconstructions, as well as 159 soft tissue reconstructions for soft tissue coverage (96 pedicle flaps, 22 free tissue transfer, 41 skin-/mesh-graft).

Furthermore, there were 29 sequelae of first treatment (e.g., cementation).

A detailed summary of resected and reconstructured structures is provided in Table 2.

## 4. Discussion

In this article, the authors describe the surgical spectrum of a sarcoma surgeon and provide a web-based means to assess it using a structured interoperable RWDT format. Our group has recently published an article detailing the quality indicators for sarcoma care in a multidisciplinary setting, as well as introducing an interoperable digital platform capable of assessing harmonized, structured data [6]. Achieving global harmonization and scalability of medical data is a crucial step towards achieving precision medicine. Specifically, in this study, our research has focused on the surgical aspects of sarcoma care, which have been integrated into the aforementioned digital platform. The presented parameters include information on patient exposure, tumor characteristics, the surgical setting, and surgical techniques. Such information ultimately allows the definition of the complexity or even the quality of a surgical procedure within an MDT. This will be an important step to establish a new ecosystem to meet the challenges of the precision medicine era [6].

Outcome prediction in medicine with the help of digital transformation and artificial intelligence opportunities will dramatically revolutionize our current treatment approach, but it will largely depend on the availability of structured data sets [17,19]. However, because of the scarcity of sarcomas, and to be able to compare on a large scale at the international level, we need to establish a common language of exchange among experts for data harmonization. It is not enough, for example, to bundle an outcome analysis of all megaprostheses independent of their anatomic localization and (neo-)adjuvant treatments. It is necessary to focus a large-scale analysis on a specific region or clinical circumstances to determine the advantages of subtle differences. The challenge for shared sarcoma care is to, nevertheless, have adequate numbers for an analysis. We therefore need a refined interoperable digital system which allows not only a detailed assessment but also the ability to make comparisons on a large, global scale to compensate for low volume numbers which are inherent with sarcomas. The interoperable digital data platform presented herein may offer a first step in this direction.

Sarcoma surgery meets two great challenges. A sarcoma surgeon has to be technically very skillful and versatile but also needs to have a great understanding of biology. These aspects need to be reflected when the spectrum of sarcoma surgery is assessed. Therefore, we created four main groups. Obviously, from the technical aspects, all specific types of resections and reconstructions matter and are important and need to be reflected in detail in such assessment. Furthermore, the types of tumors, as well as the anatomic regions where the tumors are located, must be reflected as well. We also included indications for surgery and the involved disciplines [18]. The latter is considered important to foster interdisciplinary exchange and to respect increasing technical complexities. Obviously, the current suggestion of surgical exposure presented herein is not comprehensive and may be regularly updated, similarly to how sarcoma pathologists update their WHO classification.

For the resection of sarcomas, the anatomic localization and the biology of the tumor are critically important to define the resection planes. To achieve an oncological and functional outcome in the patient’s best interest, it is critically important that sarcoma surgery is carried out with considerations for both biological and technical principles [20,21]. To obtain and improve the biological understanding of these tumors, participation at a weekly MDT’s meeting probably represents the minimal requirement because it increases the exposure to the thinking and approach of other disciplines. Our data, for example, show that interpretations of surgical margins—i.e., how wide is wide?—may continue to vary greatly without universal harmonization of assessment. The surgeons may interpret the margin differently among themselves, but their interpretations may also differ from those of pathologists. This has great consequences for the interpretation of any comparative study and must be addressed. Modern sarcoma surgery [1,9,10,16,22,23], therefore, fosters transdisciplinary collaboration under the direction of surgeons who have a broad biological knowledge and are able to organize a team of surgeons with broad technical skills depending on the anatomic site of the tumor, which is particularly important for reconstruction after tumor resection.

Sarcoma surgery is a critical determinant for a successful treatment and outcome in sarcoma patient care. The German Cancer Society (DKG) defines in their guidelines the minimal surgical interventions per year (*n* = 15), as well as those in a lifetime experience (*n* = 50) for the sarcoma surgeon [24]. The Musculoskeletal Tumor Society—MSTS also reported the number of sarcoma surgeries performed per surgeon per year, averaging approximately 35 cases [25]. Although the number of treated patients is important, it is not discriminative enough to determine the entire spectrum of surgical exposures, as sarcoma surgery includes a wide spectrum both anatomically and biologically. The French sarcoma group nicely showed that although the absolute number of performed surgeries is important, the most important discriminator for outcome is the embedding of the surgery within an MDT [2]. This is further confirmed by Baum et al. who questioned the policy of volume-based case thresholds for complex cancer surgeries by reporting risk-standardized mortality rates to be a superior metric of surgical quality compared to volume-based metrics [3,26]. Defining the complexity of surgical procedures will, therefore, be a helpful tool to meet the requirements of the precision medicine era [11,12,18,23,27].

This study has a few limitations. The overall numbers included herein may still be relatively small and concern only one sarcoma surgeon. However, considering the yearly surgical exposure proposed by MSTS or DKG [25,28], the numbers presented herein qualify for a high-volume surgeon as per definition. Furthermore, the data presented herein are considered a starting point which needs to be elaborated on, first to discuss the parameters and then to include data from many sarcoma surgeons globally. Because this RWDT-interoperable digital platform is web-based, any surgeon can store the personal information anonymously within this interoperable digital platform for free, which makes it possible to collect a vast spectrum of information.

## 5. Conclusions

The spectrum of sarcoma surgery not only is defined by surgical, technical, and biological skills but also critically depends on the integrated understanding of an orchestrated transdisciplinary treatment approach together with non-surgical disciplines. The multidisciplinary team meeting is an integral part of sarcoma surgery. If we aim at improving the quality of sarcoma patient care, it is time to move beyond assessing the raw numbers of surgeries performed. The definition of the quality ultimately assumes the comprehensive assessment of all important transdisciplinary parameters with the help of an interoperable digital platform. If the MDT is accepted as the key component for delivering high-quality care, such a platform has to reflect the interplay of disciplines, which then needs to be expelled as such to meet the precision medicine requirements. In a first step, global harmonization of data assessment on a large scale represents the prerequisite.

## Figures and Tables

**Table 1:** This summary of all tumors included in the analysis over a 10-year period is split according to the diagnoses of the WHO classification.

Soft Tissue	628	Bone	339
Adipocytic	258	Chondrogenic	141
Fibroblastic/myofibroblastic	97	Osteogenic	90
Undifferentiated/unclassified sarcoma	82	Tumors of undefined neoplastic nature	45
Tumors of uncertain differentiation	71	Osteoclastic giant cell rich	24
Nerve sheath tumors	49	Ewing	18
Fibro-histiocytic tumors	21	Notochordal tumors	7
Vascular tumors of soft tumors	14	Undifferentiated high-grade pleomorphic sarcoma	4
Smooth muscle tumors	19	Fibrohistiocytic	3
Chondro-osseous tumors	10	Fibrogenic	2
Pericytic tumors	4	Myogenic, lipogenic, epithelial tumors	2
Skeletal muscle tumors	4	Tumor syndromes	2
		Vascular tumors	1
Non-neoplastic/simulator	62		
Metastasis	44		
Lymphoma myeloma leukemia	12		
Sequelae of prior therapy	8		

**Table 2:** This summary provides a detailed overview of performed resections (left) and reconstructions (right).

Resection	Count	Reconstruction	Count
**Bone**	**489**	**Bone**	**319**
simple curettage	107	cementation	18
rotationplasty (lower extremity)	2	ORIF (incl. bone ankers; removal of OS material)	61
hemi-cortex resection	20	autograft	11
complete bone resection: extra-articular	108	vascularized fibula autograft (based on fibular artery)	10
complete bone resection: transarticular	92	non-vascularized fibula autograft	1
with 3D patient-specific cutting guides	23	allograft chips	45
radiofrequency ablation (RFA); cryotherapy, MR-HIFU	41	bulk allograft	32
tendon resection	2	conventional prosthesis	9
ligament resection	1	modular tumor prosthesis	79
forced epiphyseolysis OT (Canadell technique)	1	custom-made prosthesis	2
extra-articular scapulo-humeral resection (Tikhoff–Linberg)	1	growing prosthesis	4
biopsy/gain of diagnostic tissue	12	pedicle screws/rods/cages	18
removal of cement	1	other bone reconstruction	11
resection–replantation (upper extremity)	1	distraction osteogenesis	1
Internal hemipelvectomy	38	artificial bone substitute (Ca-sulfate, etc.)	7
Type I—ilium	15	cement spacer/pseudarthrosis/flail joint	2
Type II—Acetabular	13	arthrodesis	4
Type III—Pubic	4	vascularized epiphyseal transfer (based on tibial anterior artery)	2
Type IV—Sacral	6	Gore-Tex mesh, Trevira, etc.	2
Amputation	39	**Soft Tissues**	**159**
Forequarter	5	skin-/mesh-graft	41
External hemipelvectomy	5	pedicled tissue transfer	96
Upper extremity	5	rectus abdominis	3
Lower extremity	24	rectus abdominis (with skin)	7
**Soft Tissues**	**1800**	gastrocnemius	10
simple	694	latissimus dorsi	12
tendon resection	23	latissimus dorsi (with skin)	3
ligament resection	5	gracilis	3
resection of funiculus, scrotum, genitals	3	soleus	3
other STS resection	11	ALT	8
muscle resection	419	other muscle flap	47
vessel dissection	225	free tissue transfer	22
nerve dissection	270	latissimus dorsi	8
periosteum resection	41	gracilis	2
bone resection	20	ALT	8
vessel resection	38	other perforator flap	3
nerve resection	50	other free tissue transfer	1
MR-HIFU	1	**Chest wall**	**11**
**Chest/Thoracic**	**11**	**Abdomen**	**14**
chest wall resection	7	abdominal wall	4
other chest/lung resection	2	colon anastomosis	3
wedge resection	2	bladder	2
**Abdomen**	**19**	ureter	2
abdominal wall resection	1	other intraabdominal reconstruction	5
kidney	2	**Sequelae of First Treatment**	**29**
suprarenal glands	1	cement spaces implantation	4
ureter	3	partial implantation/replacement	22
bladder	3	complete compartment implantation/replacement	3
colon/rectum	4	**Neurovascular**	**70**
bowel	2	vascular	56
uterus/ovaries	1	artery complete	14
other abdominal resection	2	vein complete	13
**Sequelae of 1st treatment**	**106**	lympho-venous	21
debridement	27	other vessel reconstruction	8
inlay change	5	neural	14
partial removal of prosthesis	26	nerve reconstruction	8
complete removal of prosthesis	3	neurotization/local transfer	2
infection	7	autologous	4
wound healing breakdown	11	**Tendon/Ligament**	**38**
osteosynthesis breakdown	2	autologous tendon transfer	18
fracture	1	allograft tendon reconstruction	2
other	24	local tendon reconstruction	18

## Data Availability

The data that support the findings of this study are available on request from the corresponding author.
